# Supplementary material for: A UMLS-based spell checker for natural language processing in vaccine safety
Source: BMC Med Inform Decis Mak. 2007 Feb 12;7:3. doi: 10.1186/1472-6947-7-3 (PMC1805499; doi:10.1186/1472-6947-7-3)
Supplement: Additional file 13 — RAP application directory. Sets up directory for RAP (RDF application for PHP) source code [file 1472-6947-7-3-S13.gz › rap/doc/phpdoc/__filesource/fsource_default__vocabularyRSS_RES.php.html]

xml version="1.0" encoding="iso-8859-1"?


File Source for RSS\_RES.php


# Source for file RSS\_RES.php

Documentation is available at RSS\_RES.php

```
1. <?PHP
2. // ----------------------------------------------------------------------------------
3. // RSS Vocabulary (ResResource)
4. // ----------------------------------------------------------------------------------
5. // Version                   : 0.9
6. // Authors                   : Tobias Gau� (tobias.gauss@web.de)
7. //
8. // Description               : Wrapper, defining resources for all terms of RSS.
9. //                               For details about RSS see: http://purl.org/rss/1.0/.
10. //                                Using the wrapper allows you to define all aspects of
11. //                             the vocabulary in one spot, simplifing implementation and
12. //                             maintainence.
13. //
14. // ----------------------------------------------------------------------------------
15. // History:
16. // 11-08-2003                 : Initial version
17. // ----------------------------------------------------------------------------------


23. class RSS_RES{

25. function CHANNEL()
26. {
27. return  new ResResource(RSS_NS . 'channel');

29. }

31. function IMAGE()
32. {
33. return  new ResResource(RSS_NS . 'image');

35. }

37. function ITEM()
38. {
39. return  new ResResource(RSS_NS . 'item');

41. }

43. function TEXTINPUT()
44. {
45. return  new ResResource(RSS_NS . 'textinput');

47. }

49. function ITEMS()
50. {
51. return  new ResResource(RSS_NS . 'items');

53. }

55. function TITLE()
56. {
57. return  new ResResource(RSS_NS . 'title');

59. }

61. function LINK()
62. {
63. return  new ResResource(RSS_NS . 'link');

65. }

67. function URL()
68. {
69. return  new ResResource(RSS_NS . 'url');

71. }

73. function DESCRIPTION()
74. {
75. return  new ResResource(RSS_NS . 'description');

77. }

79. function NAME()
80. {
81. return  new ResResource(RSS_NS . 'name');
82. }
83. }


86. ?>
```

Documentation generated on Fri, 17 Dec 2004 16:18:07 +0100 by phpDocumentor 1.3.0RC3
